# Incidence and Severity of Post-dural Puncture Headache in Non-obstetric Patients Undergoing Subarachnoid Block

**DOI:** 10.7759/cureus.47442

**Published:** 2023-10-21

**Authors:** Leonor Aniceto, Luís Gonçalves, Lucia Gonçalves, Rita Alves, Décia Gonçalves, Marta Laranjo, Elisabete Valente

**Affiliations:** 1 Anesthesiology, Centro Hospitalar de Leiria, Leiria, PRT

**Keywords:** regional anesthesia, incidence, subarachnoid block, non-obstetric, post-dural puncture headache

## Abstract

Background and goal of the study

Post-dural puncture headache (PDPH) is a complication of central neuraxial block, either subarachnoid block (SAB) or epidural block. This clinical entity has a wide incidence and is affected by factors such as age, gender, needle gauge, needle shape/type, number of puncture attempts, and previous history of headache. Due to the lack of data in the non-obstetric population, this study assesses the incidence and severity of PDPH after SAB.

Materials and methods

A prospective observational study was carried out on patients undergoing SAB during the last trimester of 2020. Data were recorded on the day of surgery, 48 hours, and seven days after surgery. Data collected included demographic and medical clinic information, SAB procedure details, and clinical outcomes related to the presence of PDPH.

Results and discussion

Overall, 143 patients were included (median age: 62 years; 53.1% were women (n=76)). Most patients were aged >60 years (55.9%; n=80) and ASA 2 classification (65.0%; n=93). The incidence of PDPH was 21.7% (n=31), and most cases were from inpatient surgery (58.3%, n=21). The incidence of PDPH was 2.5 times higher with the use of 25 gauge compared to the use of the 27 gauge needle and was more prevalent with the use of the Quincke needles.

Conclusion

Over 20% of patients undergoing SAB experienced PDPH. Previous history of headache, larger gauge, and the Quincke needle use were associated with a higher incidence of PDPH.

## Introduction

Post-dural puncture headache (PDPH) is a complication of central neuroaxis block, which can occur after subarachnoid block (SAB) or inadvertent dural tap in epidural block [1-5]. It presents as a headache with postural characteristics (that is worse with sitting or standing and relieved with dorsal decubitus), with the onset of symptoms usually 48-72 hours after dural mater puncture and although spontaneous resolution can occur, PHDH needs proper pharmacological or procedural interventions [2,4,5]. The headache may be localized in the frontal, occipital, or both regions and is associated with symptoms such as neck stiffness, photophobia, nausea, or tinnitus [1-5].

The etiology of PDPH is not fully understood [2-7]. It is thought to be related to the loss of cerebrospinal fluid (CSF) through the hole in the dura mater secondary to the neuraxial technique, where CSF leaks. This mechanism leads to hypotension of the fluid, which is compensated with meningeal vasodilation associated with stretching of the intracranial nervous structures, with consequent appearance of a headache [4-7].

The incidence of PDPH is variable, ranging from 3 to 40% [1,3,8,9]. Young patients, female gender, and low body mass index are associated with increased risk. Other factors, such as pregnancy, previous history of headache, needle gauge and needle type, number of puncture attempts and the experience of the anesthesiologist, affect the incidence of PDPH [4,10].

Recommendations such as bed rest, hydration, or caffeine consumption are often used as prophylactic or therapeutic measures, despite the literature not being consensual in their use [8,9,11,12].

Given the lack of data and studies regarding the incidence of PDPH in non-obstetric population undergoing SAB, the working group set out to assess the incidence and severity in patients who underwent scheduled or urgent SAB in the operating room of Leiria’s Hospital (Centro Hospitalar de Leiria [CHL]) in Portugal.

This article was previously presented as a meeting abstract at Euroanaesthesia 2023 in Glasgow, on June 4, 2023.

## Materials and methods

This was a prospective observational study conducted in patients undergoing SAB at CHL, proposed for elective (inpatient and outpatient) or urgent surgery (including Orthopedics, General Surgery, Urology and Gynecology), between October and December 2020.

The primary goal was to calculate the incidence of PDPH (defined as a headache presenting within seven days after a SAB, with positional characteristics and with or without associated symptoms [13]) in patients undergoing SAB. The secondary objectives were to assess the severity of PDPH seven days after procedure, identify the predictors of PDPH, and analyze the response to PDPH treatment.

Inclusion criteria were age (18 years or older), written informed consent and SAB as the anesthetic technique used and exclusion criteria included patient refusal to participate in the study, contraindications for SAB (patient refusal, coagulopathy, thrombocytopenia <80 000 per microliter of blood, local infection, sepsis, severe hypovolemia [13,14]), dementia, death, and application of another neuroaxis anesthetic technique (including epidural or sequential).

During the course of SAB, all patients were monitored according to the American Society of Anesthesiology (ASA) standards and had at least one peripheral venous access which allowed for a balanced solution perfusion before the anesthetic technique. Lumbar puncture was performed between the intervertebral spaces of L3-L5, in the lateral decubitus or sitting positions. A volume of local anesthetic (bupivacaine or levobupivacaine) was administered to all patients to achieve a sensory block at the level of the T6-T10 dermatome before surgical incision.

Data were collected at three different time points: at the time of SAB, 48 hours post-procedure (Assessment 1) and day 7 post-procedure (Assessment 2).

At the time of the procedure, the anesthesiologist responsible for performing the technique collected the data: gender, age group, ASA classification, previous history of headache, surgical specialty, elective or urgent surgery, needle characteristics, such as bevel type (Quincke or Whitacre), gauge (25G or 27G) number of punctures, patient positioning (sitting or lateral decubitus), type of approach (median or paramedian), pain experienced by the patient during the technique, local anesthetic (LA) and opioid used, and who performed the technique (specialist, resident or both).

Data collection in Assessment 1 and 2 was performed at a follow-up in-person appointment or phone call and included presence and location of the headache (occipital, temporal or frontal), pain assessment using the numerical scale (NS 1-3: mild pain; 4-6 moderate pain; 7-10 severe pain), associated symptoms (nausea and/or vomiting, dizziness, tinnitus, neck stiffness, puncture site pain), therapy instituted (conservative, invasive or other), and clinical status after treatment (no improvement, improvement or worsening of symptoms). During Assessment 2, in addition to the aforementioned data, the following were added: onset of symptoms of PDPH for those with onset after 48h and need for medical care.

Clinical severity was defined as the presence of moderate-severe headache with/without associated symptoms, the need to visit the emergency department/attending physician, or the use of invasive techniques for treatment. Conservative treatment included oral analgesia (paracetamol, combination of paracetamol and caffeine), hydration, and bed rest in the supine position [11]. Invasive treatment included the use of blood patches [15,16].

Categorical variables were described using relative (%) and absolute (n) frequencies, while numerical variables were summarized using measures of central tendency (mean and median) and dispersion (minimum, maximum and standard deviation). The results of the categorical variables in studied were compared using the Chi-square and Fisher’s Exact Tests. The statistical treatment of the data was performed using R.

This study was approved by the Ethics Committee of CHL. In addition to obtaining written informed consent from all participants, data confidentiality was ensured throughout the process.

## Results

Demographic and perioperative characteristics

Overall, 143 non-obstetric patients underwent SAB in the CHL operating room participated in this study, as shown in Table 1. Females were the most frequent group (53.1%; n=76) as well as ASA 2 classification (65.0%; n=93), and the median of age was 62 years (55.9% [n=80] aged higher than 60 years). Most patients (62.94%; n=90) underwent surgery on an outpatient basis, with general surgery (51.05%; n=73) being the most representative medical specialty.

**Table 1:** Demographic and perioperative characteristics of the study population NS: Numerical scale; PDPH: post-dural puncture headache

			Total of cases	Cases with PDPH		p-value
Variable	Category	Absolute frequency (n)	Relative frequency (%)	Absolute frequency (n)	Relative frequency (%)	
Total		143	100%	31	21.7%	
Gender	Female	76	53.1%	20	26.3%	p = 0.374
	Male	67	46.9%	11	16.4%	
Age Group	20-40	15	10.5%	4	26.7%	p = 0.008
	41-60	48	33.6%	17	35.4%	
	>60	80	55.9%	10	12.5%	
ASA	1	13	9.1%	3	23.1%	p = 0.135
	2	93	65.0%	25	26.9%	
	3	36	25.2%	3	8.3%	
	4	1	0.7%	0	0.0%	
Regimen	Inpatient	36	25.2%	21	58.3%	p = 0.262
	Outpatient	90	62.9%	9	10.0%	
	Urgent	17	11.9%	1	5.9%	
Specialty	General surgery	73	51.0%	13	17.8%	p = 0.046
	Orthopaedics	28	19.6%	5	17.9%	
	Gynecology	30	21.0%	12	40.0%	
	Urology	12	8.4%	1	8.3%	

Thirty-one patients (21.7%) presented with PDPH. The prevalence of PDPH was higher in patients aged 41-60 compared with other age groups (p=0.008). The occurrence of PDPH was also higher in the gynecology group (p=0.046).

The SAB technique was mostly performed with a Quincke needle (65.7%; n=94) and 25G was the most frequent gauge used (76.2%; n=109). The paramedian neuraxial approach was applied in 58% (n=83) of the patients, and the most common position was lateral decubitus (93.7%; n=134), as shown in Table 2. Among the 31 patients who had PDPH, all were had less than three punctures.

**Table 2:** Characteristics of SAB techniques performed in the study population PDPH: Post-dural puncture headache; SAB: subarachnoid block

		Total		Cases with PDPH		p-value
Variable	Category	Absolute frequency (n)	Relative frequency (%)	Absolute frequency (n)	Relative frequency (%)	
Needle type	Quincke	94	65.7%	20	21.3%	p = 0.872
	Whitacre	49	34.3%	11	22.4%	
Needle gauge	25	109	76.2%	22	20.2%	p = 0.478
	27	34	22.8%	9	37.5%	
Technique approach	Median	60	42%	11	18.3%	p = 0.538
	Paramedian	83	58%	20	24.1%	
Positioning	Sitting	9	6.3%	2	22.2%	p = 0.869
	Lateral decubitus	134	93.7%	29	21.6%	
Number of puncture	<3	125	87.4%	31	24.8%	p = 0.029
	≥3	18	12.6%	0	0.0%	
Pain	Yes	5	3.5%	0	0.0%	p = 0.218
	No	138	96.5%	31	22.5%	
Blood on the needle	Yes	5	3.5%	0	0.0%	p = 0.676
	No	138	96.5%	31	22.5%	

In the group who presented PDPH, six patients (19.4%) had a previous history of headache, and there was a statistically significant association between these two variables, as shown in Table 3.

**Table 3:** Characteristics of cases with PDHP NS: Numerical scale; PDPH: post-dural puncture headache

		Absolute frequency (n)	Relative frequency (%)	p-value
Cases with PDPH		31	100.0%	
Previous history of headache		6	19.4%	p = 0.001
NS	1 - 3 Mild	17	54.8%	
	4 - 6 Moderate	7	22.6%	
	7 - 10 Severe	6	19.4%	
	Unknown	1	3.2%	
Location of headache	Frontal	25	80.6%	
	Occipital	5	16.1%	
	Temporal	0	0%	
	Unknown	1	3.2%	
Associated symptons	Yes	15	48.4%	
	No	16	51.6%	
Type of symptoms associated	Tinnitus	3	9.7%	
	Nausea and/or vomiting	1	3.2%	
	Vertigo	7	22.6%	
	Pain at puncture site	3	9.7%	
	Stiff neck	2	6.5%	
	Pruritus	1	3.2%	
Treatment	Oral Hydration + bed rest	1	3.2%	
	Paracetamol	22	71.0%	
	Paracetamol + caffeina	6	19.4%	
	Blood patch	1	3.2%	
	Unknown	1	3,2%	
Clinical status after treatment	Improved	28	90,3%	
	Mantained	2	6,5%	
	Unknown	1	3,2%	

Most patients presented with mild PDPH (NS 1-3/10) (54.8%; n=17). Headache was frontal in 25 cases (80.6%) and 15 patients had some type of associated symptoms (48.4%). In this group, vertigo was the most common presentation (22.6%; n=7), followed by tinnitus and pain at the puncture site with an equal percentage (9.7%; n=3).

All patients with PDPH were initially treated conservatively, and 90.3% of cases (n=28) had clinical improved. It should be noted that two patients maintained clinical status after conservative treatment (Table 3). One patient received blood patch and improved after this procedure.

## Discussion

The study investigated the incidence of PDPH and related factors in a non-obstetric patient population. The key findings are as follows: The incidence of PDPH in the study population was 21.7%, which falls within the range reported in the literature (4-30%) [1,3,4,8,10,17-19]. Notably, the wide range of incidence rates in previous studies may be attributed to variations in needle types and gauges, particularly in studies involving pregnant women.

Contrary to some literature suggesting a higher incidence in younger individuals, this study found that PDPH was more common in older age groups, with the majority of patients being over 40 years old. This difference may be due to the focus of previous studies on pregnant patients, who tend to be younger [19].

The incidence of PDPH varied by surgical specialty, being higher in gynecological procedures (40%; n=12-9) and lower in urology (8,3%; n=1). However, the sample size in some specialties was limited, suggesting the need for further studies with broader representation.

According to the literature, the gauge, the needle type, and the number of puncture attempts are considered modifiable risk factors for the occurrence of PDPH [6]. The choice of needle gauge appeared to influence PDPH incidence, which was higher with the use of Quincke needles, but the difference was not statistically significant [1,4,5,20-22]. Nevertheless, smaller gauge needles, while reducing PDPH risk, can pose technical challenges leading to multiple puncture attempts [4,23].

Inpatient procedures had a significantly higher incidence of PDPH compared to outpatient procedures. At an organizational level, it may be relevant to re-evaluate the anesthetic approach used in inpatient, given the impact of PDPH on the length of hospital stay, diagnostic tests and treatment and the significant financial repercussions associated [1].

Surprisingly, there were no cases of PDPH when the technique required more than three puncture attempts. This finding contradicts the prevailing literature, which suggests that multiple puncture attempts are associated with a higher risk of PDPH [4,8,24,25]. Differences in the definition of multiple attempts and study designs may explain this discrepancy.

Six out of the 31 patients with PDPH had a prior history of headache. The literature on this topic is diverse, with variations in how previous headache and PDPH are differentiated [6,8,14].

PDPH occurrence was slightly higher when a paramedian approach was used compared to the median alternative. The position of patients during the procedure was not consistently reported in similar studies, suggesting a need for future investigations [20].

Most cases of PDPH were managed conservatively, in line with the literature [2,8,11]. However, one severe case required invasive treatment with an epidural blood patch, resulting in symptom resolution [9,20]. One patient missed the follow-up.

The study's limitations include a limited range of surgical specialties and the omission of certain variables that could impact PDPH incidence, such as dehydration level, body mass index, needle orientation, and puncture level. More studies in the non-obstetric population and investigations considering similar variables are suggested to better understand risk and protective factors related to PDPH in diverse patient populations.

## Conclusions

More than 1 in 5 patients undergoing SAB in the non-obstetric population had PDPH in our study and previous history of headache, larger gauge needle and the use of Quincke type needle are associated with higher incidence of PDPH. In most cases of PDPH, a conservative treatment used was effective without the need for additional therapy.

In summary, while the study provided valuable insights into PDPH incidence and related factors in a non-obstetric patient population, its limitations, including the limited scope of surgical specialties and missing variables, should be considered when interpreting and applying the findings. Further research with a more comprehensive approach and larger sample sizes may help address some of these limitations.
